# Effects of computer familiarity and computer type on the performance of Korean computerized neurobehavioral test

**DOI:** 10.1186/s40557-016-0129-9

**Published:** 2016-09-09

**Authors:** Nak Joon Baek, Gun Il Park, Young Seok Byun, Man Joong Jeon, Joon Sakong

**Affiliations:** 1 Department of Occupational and Environmental Medicine, Yeungnam University Hospital, 170 Hyeonchung-ro, Nam-gu, Daegu, 42415 Republic of Korea; 2 Department of Preventive Medicine and Public Health, College of Medicine, Yeungnam University, 170 Hyeonchung-ro, Nam-gu, Daegu, 42415 Republic of Korea

**Keywords:** Neurobehavioral, Familiarity, Type of keyboard, Type of computer

## Abstract

**Background:**

It is thought that computer familiarity has increased significantly since 2004 as well as the use of computers. This study aimed to evaluate the effects of computer familiarity and types of keyboard and computer on the performance of the Korean computerized neurobehavioral test (KCNT), and to identify which parameters of KCNT were affected by aforementioned factors.

**Methods:**

A total of 85 subjects were classified into three groups of computer familiarity by Korean typing speed. Their age, gender and the level of education were also collected. The parameters of KCNT included simple reaction time, choice reaction time, addition, symbol digit, and finger tapping speed. The test was conducted using three types of computers: a laptop computer, a laptop computer with a simplified keyboard, and a desktop computer with a simplified keyboard.

**Results:**

Parameters including the simple reaction time, choice reaction time, addition, and symbol digit, and the finger tapping speed of non-dominant hand showed no significant differences in the results among the three groups by computer familiarity after age and educational years were controlled as covariates. The mean reaction time of the simple reaction time and the choice reaction time with a simplified keyboard was significantly shorter compared to that with a typical keyboard. With regard to type of computer, the mean reaction time of the simple reaction time and the choice reaction time was significantly reduced when performed with the desktop computer with a simplified keyboard.

**Conclusions:**

Unlike previous study results, the choice reaction time, the addition, and the finger tapping speed of dominant hand were the only parameters affected by the computer familiarity. Both the type of keyboard and the type of computer significantly influenced the simple reaction time and the choice reaction time. Therefore, it is recommended to use a desktop computer with a simplified keyboard for such parameters.

## Background

The neurobehavioral test is used to evaluate the hazard of neurotoxic materials such as heavy metals including manganese and organic solvents on the nervous system [1–5]. For the neurobehavioral test, various traditional and computerized tests are used, and these are employed as a form of battery to test the neurobehavioral performance in many fields [6–10].

According to the study of Chung et al. [7], parameters of the computerized neurobehavioral test including symbol digit, finger tapping speed showed higher validities than those of the traditional tests. Additionally, in the study of Sakong et al. [8, 9] that assessed the retest reliability coefficients of the neurobehavioral test, among the parameters of the computerized neurobehavioral test, addition, finger tapping speed, symbol digit, and digit span showed higher reliability coefficients, whereas in the traditional neurobehavioral test, only digit symbol and pursuit aiming had appropriate reliability coefficients. Consequently, the study recommended that the computerized neurobehavioral test be preferentially considered when a neurobehavioral test battery is constructed because the computerized tests had a higher reliability.

The greatest advantage of the computerized neurobehavioral test is that the test and reaction processes are standardized. Namely, testing and scoring processes are standardized in the computerized neurobehavioral test. Therefore, comparison of results is easy; each stimulus and reaction can be observed individually; the performance can be scored; and saving and transforming data are easy. Moreover, the test can be processed by a nurse or a technician, parameters can be promptly selected or changed according to the characteristics of the workers, and it is easy to apply in the workplace because the test is movable [7–10].

Among computerized neurobehavioral tests, the Korean Computerized Neurobehavioral Test System (KCNT) that is used as a secondary optional test in the special periodic health examination was developed in Korea and has been utilized since 2009. The test can be conducted when nervous system disorders are concerned due to the exposure to organic compounds such as chloromethane, 1,2-dichloroethylene, methyl alcohol, methyl ethyl ketone, methyl isobutyl ketone, 2-bromopropane, methyl bromide, carbon tetrachloride, styrene, acetone, acetaldehyde, acrylonitrile, acrylamide, ethylene glycol, ethylene dichloride, carbon disulfide, xylene, chlorobenzene, tetrahydrofuran, toluene, methenyl trichloride, n-hexane, and hydroquinone; metals such as lead and it’s compound, and manganese and it’s compound; gaseous substances such as hydrogen cyanide, and nitrogen monoxide; and physical factors such as micro-wave, radio-wave, etc. [11].

However, in the computerized neurobehavioral test, the types of stimuli are limited to visual ones via the computer monitor, and the ways of reactions are also restricted by an input tool such as keyboard, or joystick [10, 12–14]. Therefore, there is a difference in familiarity with the computer between subjects who have frequently contacted a computer or video games and subjects who have not, which may cause an error in the computerized neurobehavioral test. Particularly, the effect of an input tool, a keyboard, can be considered, and the result of evaluating the effect of the type of keyboard on test results showed that for the parameters such as symbol digit that may be affected by computer familiarity and the type of keyboard, using a simplified keyboard after leaving the keys necessary for the computerized neurobehavioral test was helpful to obtain more valid test results than when a typical full key keyboard was used [10].

In addition, when many workers need to be examined, it is inconvenient to move and install many desktop computers for the test, thus if laptop computers convenient in carrying and installing are used, the inconvenience caused when ordinary desktop computers are used will be reduced. With regard to this, in 2004, the computerized neurobehavioral tests were performed using three types of computers (laptop computer, laptop computer equipped with the simplified keyboard, ordinary desktop computer equipped with the simplified keyboard). The study reported that using a laptop computer only was inappropriate, and using a laptop computer equipped with the simplified keyboard or an ordinary desktop computer equipped with the simplified keyboard was helpful to obtain more valid test results [15].

Meanwhile, in 2004 when the previous study on the computerized neurobehavioral performance by the type of keyboard and by the type of computer had been conducted, the distribution rate of the desktop computer per household in Korea (unit: computer/ household) was 0.77, while that of the laptop computer was 0.05. However, in 2011, the distribution rate of the desktop computer changed to 0.75, and that of the laptop computer increased to 0.25. Consequently, it is thought that the familiarity of laptop computer increased in 2011 compared to that in 2004, as the distribution rate of the laptop computer has risen [16].

Therefore, this study was conducted to use the KCNT more effectively that is currently employed in the special periodic medical examination by evaluation of the effects of computer familiarity and computer type on the performance of KCNT and selection the parameters of KCNT least affected by computer familiarity and type of computer in the situation when the use of computer increased, and the degree of laptop computer use changed compared to the situation in 2004.

## Methods

### Participants

Participants were recruited from the workers who visited the hospital for a special periodic health examination and local residents. The study purpose was explained to them, then those who understood the purpose of the study and gave consent to study participation were included in this study by convenience sampling. Additionally, those who had a physical disability diagnosed by a doctor which might affect the neurobehavioral test, including hypertension, diabetes mellitus, hearing and visual impairments, head trauma and severe back pain, etc., those who had been taking a drug due to a chronic disease for a long time, and those who had been exposed to neurotoxic materials such as organic solvents, welding work, etc. were excluded. Since the simple reaction time among parameters of KCNT is considered as the most powerful and sensitive parameter to be used in screening workers of neurotoxicity, acceptable mean difference of 33 msec between groups and standard deviation of 74 msec were used to calculate the sample size. With this information we obtained a sample size of 78.85 subjects per group for a 80 % statistical power with α = 0.05. Therefore, a total of 85 persons participated, and their computerized neurobehavioral performance scores were analyzed. To enable direct comparison with previous studies conducted in 2004 under identical conditions, the same method was used to classify the participants’ familiarity to the computer into three different groups [10, 15]. After the typing speed per minute was measured using a typing practice program, the participants with a score of near-zero were assigned to the group I, no competency using computer; the participants with a score of less than 200 were assigned to the group II, relatively familiar with the computer; and the participants with a score of over 200 were assigned to the group III, very familiar with the computer. As a result, 8 persons were assigned to the group I; 34 persons to the group II; and 43 persons to the group III. The participants were requested to take a descent sleep a day before the test and to avoid taking a drug or drinking. This study was approved by the Institutional Review Board of Yeungnam University (IRB No. 7002016-A-2015-051).

### Computerized neurobehavioral performance test

The Korean Computerized Neurobehavioral Test (KCNT)® of MaxMedica Inc. based on Swedish Performance Evaluation System (SPES) was employed that was developed to be used for Korean adults and has been used since 2009 as a secondary optional test in the special periodic medical examination for Korean workers [17].

The parameters of the KCNT including simple reaction time, choice reaction time, addition, symbol digit, and finger tapping speed were tested for all subjects in the same order, and the test method and scoring process were the same as those in 2004 [10]. They are as follows.

In the simple reaction time, a red rectangle irregularly appears in the center of the computer screen with 2.5–5 s intervals. Subjects are guided to press the space bar on the keyboard as quickly as possible if a rectangle appears. The computer measures the time from the moment that a rectangle appears on the screen until a subject presses the key in the unit of 0.001 s. The rectangle appears 32 times for 2 min, and each reaction time when a rectangle appears, and the mean and standard deviation of the reaction times are automatically recorded.

In the choice reaction time, a yellow cross irregularly appears on the screen with 2.5–5 s intervals. Subjects are guided to press an arrow key with the same direction using the index finger as quickly as possible after finding the shortest arm among the four arms of the cross. The cross appears 16 times for one minute, and after one-minute practice, the 2-minute-test is performed. The reaction time for each stimulus, the mean and standard deviation of the reaction time are automatically recorded.

In the addition, three random numbers appear in the center of the computer screen for one second in the form of horizontal addition (e.g., 6 + 7 + 3). Subjects are guided to calculate as quickly as possible and enter the answer via the number keys on the keyboard using the index finger. After the first three practice tests, a total of 28 problems are given, and the reaction time for each addition, the mean and standard deviation of the reaction times, and the number of correct answers are automatically recorded.

In the symbol digit, at the top of the computer screen randomly paired symbols and the numbers from 1 to 9 appear and at the bottom of the computer screen symbols arranged in a different order from the order at the top and 9 blanks appear. Subjects are guided to enter numbers in the blanks to make them consistent with the pairs of symbols and numbers presented on the top using number keys. After the first nine practice tests, a total of 36 symbol digit tests are conducted, and the reaction time for each pair, the mean and standard deviation of the reaction times, the reaction time for correct pairs, the mean and standard deviation of the reaction times of correct pairs, and the number of incorrect typing are automatically recorded.

The finger tapping speed is a test to evaluate a persistent motor ability. Subjects are guided to tap the key using the index finger as quickly as possible while putting the palm on the table. The dominant hand that is frequently used and the non-dominant hand that is not frequently used are tested by turns. After one time practice for each hand, the test is given twice for each hand, and the computer records the number of tapping for 10 s, the mean numbers of tapping of both hands, and standard deviation of the numbers of tapping.

### Types of using computer and keyboard

Like the study performed in 2004, three types of computers were used: type I, laptop computer; type II, laptop computer equipped with a simplified keyboard; and type III, desktop computer equipped with a simplified keyboard. To reduce the effect of keyboard, a simplified keyboard was employed, and validity of which has been demonstrated by Jeon et al. [10] prior to its application in the study of Kim et al. [15], where test performances by the type of computer were compared. Namely, a simplified 17-key keyboard was used where unnecessary keys other than 17 keys essential for the test (10 number keys, 4 arrow keys, both Ctrl keys, and a space key) were all removed and completely hidden by a hard board cover. The size of monitor of the desktop computer that was used in the test was 22 in., and its aspect ratio was 16:9, and that of the laptop computer was 15.4 in. and its aspect ratio was 4:3.

### Data collection

The subject's gender, age, and the level of education were examined, and the KCNT was conducted. The test was performed by a doctor experienced in the KCNT in the same place for all subjects, which was quiet and relatively isolate. The test was performed one subject at a time, and based on the instruction prepared in advance, subjects were educated in the same way regarding the test process of each parameter, the type of stimulus, and how to react to the stimulus using the keyboard. If a subject had a hard time understanding, additional explanation was given by an examiner. By doing this, the conversation between the examiner and examinee was standardized and minimized. Furthermore, when testing subjects using the three different types of computers sequentially, six possible combinations of the order were randomly assigned to each subject to minimize the learning effect and errors in interpretation of results.

### Statistical analysis

The results were analyzed using the IBM SPSS Statistics (version 22) program. To compare age, educational years, typing speed, and KCNT results among three types of computers, one way ANOVA was used, and to examine a difference in the gender composition, Fisher’s exact test was used. As a post hoc test of the one way ANOVA, Bonferroni F test was used, and for nonparametric testing, Kruskal Wallis test was performed. Additionally, to compare the results of the KCNT between type I computer and type II computer, *t*-test was used, and for nonparametric testing between two groups, Mann Whitney *U* test was conducted. The KCNT results of the three groups classified by typing speed were assessed by ANCOVA in which age and educational years were controlled as covariates. The significance level was determined to be *p* < 0.05.

## Results

### Demographic data

The mean age of subjects was 37.5 ± 13.3 years. Subjects were classified into group I, II, and III by computer familiarity based on the Korean typing speed in the same way that was used in the previous study [10] performed in 2004. The mean age was 58.6 ± 4.3 years in the group I, 41.1 ± 11.4 years in the group II, and 30.6 ± 10.1 years in the group III. The differences in age among the three groups were significant (*p* < 0.001). There was no difference in the male-female composition among the three groups. The mean education years of the group I was 13.3 ± 3.5 years, which was significantly lower than 15.5 ± 1.5 years in the group II and 15.1 ± 1.8 years in the group III (*p* = 0.011). The typing speed per minute was 0 in the group I, 119.7 ± 65.2 in the group II, and 325.4 ± 110.9 in the group III. The differences among the three groups on the Korean typing speed were significant (*p* < 0.001) (Table 1).

**Table 1:** General characteristics of the subjects

Characteristics	Group I (*n* = 8)	Group II (*n* = 34)	Group III (*n* = 43)	*p*-value^*^
Age (years)	58.6 (4.3)^a^	41.1 (11.4)^b^	30.6 (10.1)^c^	< 0.001
Sex *(n* (%))				
Men	6 (75.0)	23 (67.6)	24 (55.8)	0.477
Women	2 (25.0)	11 (32.4)	19 (44.2)	
Education (years)	13.3 (3.5)^a^	15.5 (1.5)^b^	15.1 (1.8)^b^	0.011
Typing speed^†^	0^a^	119.7 (65.2)^b^	325.4 (110.9)^c^	< 0.001

The value are expressed as mean (standard deviation) for age, education, and typing speed

Group I, subjects with a score of 0 per minute (no competency using computer); Group II, subjects with a score of less than 200 per minute (relatively familiar with the computer); Group III, subjects with a score of over 200 per minute (very familiar with the computer). *calculated by one-way ANOVA or Fisher’s exact test, different letters such as ^a, b^ and ^c^ in row indicate differences at the 5 % significance level by post hoc test of Bonferroni F. ^†^number of Korean character typed in a minute

### Comparison of the KCNT performance depending on the Korean typing speed

The results of KCNT of three groups classified by the Korean typing speed were compared. In epidemiological research or studies investigating a difference between groups with regard to the computerized neurobehavioral test, if there are differences in age and level of education between the groups, the variables need to be controlled because they have been reported as factors that affect the neurobehavioral test the most. Like the study of Jeon et al. [10], the differences in age and educational years were significant among the three groups classified by the Korean typing speed in this study. Thus age and educational years were controlled as covariates for comparison of test results among the three groups classified by the Korean typing speed in this study.

After age and educational years were controlled as covariates, in simple reaction time, there were no significant differences in the mean reaction time and the standard deviation of mean reaction times among the three groups classified by the Korean typing speed.

In choice reaction time, the three groups did not show a significant difference in the mean reaction time analyzed. But the standard deviation of its mean reaction times was significantly increased in the group I (*p* = 0.002). When tested with a desktop computer equipped with a simplified keyboard, the result was even more increased in the group I compared to the group II and the group III (*p* = 0.004).

In terms of addition, there were no significant differences in the mean reaction time and the standard deviation of the mean reaction times among the three groups. The number of errors in the group III was significantly greater than those of the group II (*p* = 0.016) (Table 2).

**Table 2:** Neurobehavioral test scores by typing speed groups

Neurobehavioral tests	Typing speed			*F*-value	*p*-value^*^
	Group I	Group II	Group III		
Simple Reaction time (msec)					
Mean reaction time	369.3 (15.5)	379.6 (5.9)	384.2 (5.8)	0.365	0.694
Computer type I	391.9 (24.8)	413.5 (9.4)	420.7 (9.4)	0.504	0.606
Computer type II	381.9 (23.6)	391.6 (8.9)	397.2 (8.9)	0.179	0.837
Computer type III	334.2 (15.7)	333.7 (6.0)	334.8 (5.9)	0.009	0.992
SD of mean reaction time	57.8 (5.4)	50.7 (2.0)	53.5 (2.0)	1.172	0.312
Computer type I	52.3 (10.3)	57.1 (3.9)	55.6 (3.9)	0.131	0.877
Computer type II	58.1 (8.2)	47.3 (3.1)	51.9 (3.1)	1.253	0.291
Computer type III	63.1 (9.6)	47.8 (3.6)	53.1 (3.6)	1.598	0.209
Choice Reaction Time (msec)					
Mean reaction time	600.2 (24.0)	604.0 (9.2)	598.6 (8.9)	0.091	0.913
Computer type I	619.8 (45.0)	649.8 (17.3)	647.5 (16.8)	0.205	0.815
Computer type II	612.5 (34.7)	612.2 (13.3)	612.7 (12.9)	0.001	1.000
Computer type III	568.2 (30.2)	550.0 (11.6)	535.7 (11.3)	0.556	0.576
SD of mean reaction time	123.7 (8.9)^ab^	90.4 (3.4)^a^	90.2 (3.3)^b^	6.385	0.002
Computer type I	113.2 (16.9)	99.3 (6.5)	95.2 (6.3)	0.425	0.655
Computer type II	127.5 (15.8)	90.0 (6.0)	93.3 (5.9)	2.628	0.079
Computer type III	130.3 (13.5)^ab^	81.9 (5.2)^a^	82.0 (5.0)^b^	5.871	0.004
Number of error	0.6 (0.3)	0.4 (0.1)	0.6 (0.1)	0.957	0.386
Computer type I	0.8 (0.8)	0.5 (0.3)	1.0 (0.3)	0.510	0.602
Computer type II	0.7 (0.3)	0.2 (0.1)	0.3 (0.1)	1.037	0.359
Computer type III	0.3 (0.3)	0.3 (0.1)	0.4 (0.1)	0.176	0.839
Addition (msec)					
Mean reaction time	2514.6 (122.1)	2506.2 (46.8)	2344.3 (45.3)	2.894	0.057
Computer type I	2517.3 (219.9)	2591.8 (84.3)	2464.1 (81.6)	0.589	0.557
Computer type II	2571.1 (226.6)	2469.3 (86.9)	2286.3 (84.1)	1.194	0.308
Computer type III	2455.5 (190.6)	2457.5 (73.1)	2282.5 (70.7)	1.382	0.257
SD of mean reaction time	494.7 (67.2)	637.1 (25.8)	613.0 (24.9)	2.205	0.112
Computer type I	506.8 (121.3)	692.1 (46.5)	646.9 (45.0)	1.248	0.293
Computer type II	487.4 (114.6)	602.8 (44.0)	570.5 (42.5)	0.571	0.567
Computer type III	489.8 (116.5)	616.3 (44.7)	621.8 (43.2)	0.549	0.580
Number of error	3.0 (0.6)	2.0 (0.2)^a^	2.7 (0.2)^a^	4.175	0.016
Computer type I	2.7 (1.0)	2.1 (0.4)	2.6 (0.4)	0.477	0.622
Computer type II	3.1 (1.0)	2.0 (0.4)	2.4 (0.4)	0.829	0.440
Computer type III	3.2 (1.0)	1.8 (0.4)^a^	3.2 (0.4)^a^	3.858	0.025

The value are expressed as mean (standard error) adjusted by age and education. *SD* standard deviation. *calculated by ANCOVA adjusted for age and education, same letters such as ^a^ and ^b^ in same row indicate differences at the 5 % significance level by post hoc test of Bonferroni F

Computer type I is laptop computer, computer type II is laptop computer connected to a simplified keyboard, and computer type III is desktop computer connected to a simplified keyboard

The mean reaction time of symbol digit did not show any significant difference among the three groups, but the standard deviation of the mean reaction times in the group I was significantly greater than that of the group III when a desktop computer equipped with a simplified keyboard was used (*p* = 0.038).

The performance of finger tapping speed with the dominant hand was the lowest in the group I (*p* = 0.012) (Table 3).

**Table 3:** Neurobehavioral test scores by typing speed groups

Neurobehavioral tests	Typing speed			*F*-value	*p*-value^*^
	Group I	Group II	Group III		
Symbol digit (msec)					
Mean reaction time	2156.3 (89.5)	2087.7 (34.0)	1974.6 (33.4)	2.930	0.055
Computer type I	2013.6 (153.2)	2126.3 (58.2)	2009.4 (57.2)	1.143	0.325
Computer type II	2217.7 (166.0)	2075.3 (63.1)	1966.9 (62.0)	1.075	0.346
Computer type III	2237.8 (150.9)	2061.5 (57.3)	1947.4 (56.3)	1.629	0.203
SD of mean reaction time	615.6 (79.0)	566.1 (30.0)	537.4 (29.5)	0.414	0.662
Computer type I	459.7 (132.4)	591.2 (50.3)	568.2 (49.4)	0.493	0.613
Computer type II	611.4 (164.5)	601.0 (62.5)	589.0 (61.4)	0.011	0.989
Computer type III	775.8 (107.0)^a^	506.0 (40.7)	455.1 (40.0)^a^	3.422	0.038
Number of error	0.2 (0.1)	0.1 (0.1)	0.1 (0.1)	0.182	0.834
Computer type I	0.0 (0.2)	0.2 (0.1)	0.1 (0.1)	1.675	0.194
Computer type II	0.4 (0.3)	0.1 (0.1)	0.2 (0.1)	0.290	0.749
Computer type III	0.2 (0.1)	0.1 (0.1)	0.0 (0.1)	2.038	0.138
Mean reaction time, correct	2154.2 (91.4)	2089.1 (34.7)	1978.6 (34.1)	2.666	0.072
Computer type I	2011.7 (153.8)	2129.0 (58.4)	2017.6 (57.4)	1.071	0.348
Computer type II	2220.5 (174.7)	2081.8 (66.4)	1969.8 (65.2)	0.998	0.374
Computer type III	2230.3 (150.6)	2056.3 (57.2)	1948.3 (56.2)	1.519	0.226
SD of mean reaction time, correct	630.0 (77.3)	550.8 (29.4)	532.7 (28.9)	0.597	0.552
Computer type I	473.7 (133.0)	590.7 (50.5)	559.3 (49.7)	0.433	0.650
Computer type II	637.0 (157.5)	561.3 (59.8)	583.5 (58.8)	0.134	0.875
Computer type III	779.3 (107.4)^ab^	500.6 (40.8)^a^	455.5 (40.1)^b^	3.508	0.035
Finger Tapping Speed^§^					
Dominant hand	58.9 (2.4)^a^	64.9 (0.9)	67.2 (0.9)^a^	4.489	0.012
Computer type I	59.6 (4.3)	63.1 (1.6)	65.8 (1.6)	0.968	0.384
Computer type II	57.2 (4.3)	65.3 (1.6)	68.4 (1.6)	2.528	0.086
Computer type III	59.7 (4.0)	66.3 (1.5)	67.3 (1.5)	1.361	0.262
Non-dominant hand	57.4 (2.4)	59.7 (0.9)	62.3 (0.9)	2.411	0.092
Computer type I	58.6 (4.2)	59.7 (1.6)	59.8 (1.5)	0.036	0.965
Computer type II	54.9 (4.2)	60.3 (1.6)	64.4 (1.5)	2.467	0.091
Computer type III	58.7 (4.2)	59.3 (1.6)	62.7 (1.5)	1.157	0.320

The value are expressed as mean (standard error) adjusted by age and education. *SD* standard deviation. *calculated by ANCOVA adjusted for age and education, same letters such as ^a^ and ^b^ in same row indicate differences at the 5 % significance level by post hoc test of Bonferroni F. ^§^parameters of Finger Tapping Speed are the number of taps for 10 s

### Comparison of the KCNT performance by the type of keyboard

To evaluate the difference in the performance of the KCNT according to different types of keyboards, the results of KCNT tested by a laptop computer alone (type I) and a laptop computer equipped with a simplified keyboard (type II) were compared. Because same participants performed all the tests with both types of keyboard, the age and level of education did not need to be adjusted for statistical analysis.

The mean reaction time of simple reaction time was 414.9 ± 53.5 msec and 393.4 ± 51.1 msec in type I and type II computers, respectively. It was significantly reduced with type II computer (*p* = 0.009). And the mean reaction time of choice reaction time was 612.5 ± 88.9 msec with type II computer which was significantly different from 645.7 ± 108.3 msec of type I computer (*p* = 0.032). But the number of errors in choice reaction time did not show any significant difference between both types of keyboards.

In addition, the mean reaction time, standard deviation of the mean reaction time, and the number of errors were not significantly different depending on the two types of the keyboard (Table 4).

**Table 4:** Neurobehavioral test scores by types of the computer

Neurobehavioral tests	Types of the computer			F or *χ* ^2*^	*p*-value^*^
	Type I	Type II	Type III		
Simple Reaction time (msec)					
Mean reaction time	414.9 (53.5)^ab^	393.4 (51.1)^ac^	334.3 (34.3)^bc^	65.181	< 0.001
Group I	412.4 (35.4)^a^	374.8 (40.6)	349.3 (31.8)^a^	6.184	0.008
Group II	417.2 (64.6)^a^	394.1 (55.7)^b^	336.1 (39.1)^ab^	20.240	< 0.001
Group III	413.6 (46.9)^a^	396.5 (49.3)^b^	330.0 (30.3)^ab^	43.288	< 0.001
SD of mean reaction time	55.9 (21.8)	50.6 (17.9)	51.9 (20.8)	1.541	0.216
Group I	55.8 (16.1)	54.1 (18.3)	62.8 (23.6)	0.439	0.650
Group II	57.7 (25.9)	48.2 (18.8)	48.8 (19.7)	2.047	0.135
Group III	54.4 (19.3)	51.9 (17.3)	52.3 (20.9)	0.202	0.817
Choice Reaction Time (msec)					
Mean reaction time	645.7 (108.3)^ab^	612.5 (88.9)^ac^	544.5 (84.9)^bc^	24.686	< 0.001
Group I	710.5 (84.2)	695.6 (108.1)	652.4 (55.3)	1.002	0.384
Group II	663.8 (119.7)^a^	624.7 (88.6)^b^	561.3 (93.5)^ab^	8.559	< 0.001
Group III	619.2 (96.2)^a^	587.1 (74.5)^b^	510.8 (58.5)^ab^	21.436	< 0.001
SD of mean reaction time	98.6 (37.4)	95.3 (36.3)	86.6 (34.4)	2.436	0.090
Group I	122.8 (35.9)	137.4 (28.5)	139.3 (33.9)	0.603	0.556
Group II	101.9 (40.4)	91.8 (38.6)	84.7 (28.6)	1.874	0.159
Group III	91.4 (33.7)	90.0 (30.8)	78.1 (30.4)	2.244	0.110
Number of error	0.8 (1.7)	0.3 (0.7)	0.4 (0.6)	3.297	0.192
Group I	0.8 (0.7)	0.6 (0.9)	0.1 (0.4)	3.757	0.153
Group II	0.5 (1.0)	0.2 (0.7)	0.3 (0.5)	1.294	0.524
Group III	1.0 (2.3)	0.3 (0.6)	0.5 (0.6)	2.493	0.287
Addition (msec)					
Mean reaction time	2520.0 (516.7)	2386.5 (516.6)	2368.8 (443.4)	2.329	0.100
Group I	2915.0 (705.7)	2807.0 (784.4)	2720.5 (673.5)	0.145	0.866
Group II	2593.2 (424.8)	2468.4 (506.0)	2462.3 (438.7)	0.858	0.427
Group III	2387.3 (505.2)	2242.1 (409.5)	2228.3 (337.8)	1.820	0.166
SD of mean reaction time	651.4 (262.5)	575.4 (243.0)	606.9 (246.7)	1.924	0.148
Group I	636.9 (253.7)	526.3 (190.6)	561.6 (249.4)	0.470	0.631
Group II	692.6 (262.7)	598.5 (245.6)	616.7 (270.2)	1.219	0.300
Group III	621.7 (265.7)	566.5 (252.7)	607.8 (231.6)	0.552	0.577
Number of error	2.4 (2.3)	2.3 (2.3)	2.7 (2.4)	1.026	0.599
Group I	4.4 (3.5)	4.4 (2.5)	4.5 (2.3)	0.188	0.910
Group II	2.2 (2.2)	2.1 (1.7)	2.1 (2.0)	0.103	0.950
Group III	2.1 (2.0)	2.1 (2.4)	2.8 (2.5)	2.391	0.303

The value are expressed as mean (standard deviation). *SD* standard deviation. *calculated by one way ANOVA or Kruskal Wallis test, same letters such as ^a^, ^b^ and ^c^ in same row indicate differences at the 5 % significance level by post hoc test of Bonferroni F

And in symbol digit and finger tapping speed, there were no significant differences in the mean reaction time, standard deviation of the mean reaction time, the numbers of errors, and the numbers of tapping between the two types of keyboards (Table 5).

**Table 5:** Neurobehavioral test scores by types of the computer

Neurobehavioral tests	Types of the computer			F or *χ* ^2*^	*p*-value^*^
	Type I	Type II	Type III		
Symbol digit (msec)					
Mean reaction time	2057.7 (395.3)	2033.9 (417.3)	2020.3 (395.0)	0.173	0.841
Group I	2437.6 (347.7)	2539.9 (452.0)	2588.4 (602.6)	0.181	0.836
Group II	2174.8 (433.2)	2127.0 (404.2)	2092.2 (335.9)	0.356	0.701
Group III	1893.5 (280.4)	1866.7 (322.8)	1859.3 (276.5)	0.146	0.864
SD of mean reaction time	567.9 (271.7)	595.9 (338.5)	504.8 (228.4)	2.120	0.122
Group I	536.4 (93.4)	700.7 (425.9)	715.4 (406.1)	0.498	0.780
Group II	588.9 (341.1)	617.5 (363.6)	501.1 (183.4)	0.618	0.734
Group III	556.3 (229.4)	559.4 (303.2)	470.0 (206.8)	2.945	0.229
Number of error	0.1 (0.4)	0.2 (0.6)	0.1 (0.2)	2.629	0.269
Group I	0.0 (0.0)	0.4 (0.8)	0.1 (0.4)	2.321	0.313
Group II	0.2 (0.5)	0.2 (0.8)	0.1 (0.3)	1.369	0.504
Group III	0.1 (0.3)	0.2 (0.4)	0.0 (0.0)	5.034	0.081
Mean reaction time, correct	2062.8 (394.5)	2038.2 (437.0)	2017.9 (394.3)	0.235	0.791
Group I	2437.6 (347.7)	2558.1 (442.8)	2585.0 (604.9)	0.190	0.829
Group II	2176.3 (437.6)	2135.9 (447.0)	2087.3 (335.3)	0.379	0.686
Group III	1902.4 (277.4)	1864.8 (323.1)	1859.3 (276.5)	0.249	0.780
SD of mean reaction time, correct	564.5 (272.4)	579.2 (323.0)	503.0 (229.6)	1.650	0.194
Group I	536.4 (93.4)	705.1 (424.2)	720.1 (403.9)	0.386	0.824
Group II	587.6 (342.8)	573.2 (319.0)	495.9 (186.6)	0.447	0.800
Group III	550.6 (228.8)	561.5 (311.0)	470.0 (206.8)	2.518	0.284
Finger Tapping Speed^§^					
Dominant hand	64.1 (9.3)	66.1 (9.5)	66.2 (9.1)	1.319	0.269
Group I	58.4 (10.3)	58.8 (10.0)	57.6 (6.9)	0.032	0.969
Group II	63.2 (8.4)	65.7 (7.2)	65.9 (7.7)	1.307	0.275
Group III	66.0 (9.4)	67.8 (10.6)	68.0 (9.6)	0.545	0.581
Non-dominant hand	59.6 (9.2)	61.8 (9.2)	60.9 (9.3)	1.235	0.293
Group I	54.1 (7.3)	55.1 (10.0)	55.3 (8.2)	0.047	0.954
Group II	59.3 (9.3)	60.2 (8.5)	58.9 (8.7)	0.214	0.808
Group III	61.0 (9.1)	64.4 (9.0)	63.6 (9.3)	1.666	0.193

The value are expressed as mean (standard deviation). *SD* standard deviation. *calculated by one way ANOVA or Kruskal Wallis test. ^§^parameters of Finger Tapping Speed are the number of taps for 10 s

### Comparison of the KCNT performance by the type of computer

Same participants were tested with all three types of computers to assess if there is any difference in the results of KCNT produced by using the computer type I, II and III. Therefore, the age and the level of education did not need to be adjusted for analysis.

The mean reaction times of simple reaction time were 414.9 ± 53.5 msec, 393.4 ± 51.1 msec, and 334.3 ± 34.3 msec with computer type I, II and III, respectively. The difference was statistically significant (*p* < 0.001). In the standard deviation of mean reaction times of simple reaction time, there was no significant difference among the three types of computers.

The mean reaction times of choice reaction time were 645.7 ± 108.3 msec, 612.5 ± 88.9 msec, and 544.5 ± 84.9 msec with computer type I, II and III, respectively. The performance of the KCNT showed significant difference in all types of computers (*p* < 0.001). And within the group II, when the type I computer was used, the mean reaction time was 663.8 ± 119.7 msec; when the type II computer was used, 624.7 ± 88.6 msec; and when the type III computer was used, 561.3 ± 93.5 msec. The difference among the three types of computers were significant (*p* < 0.001), and in the post hoc test by the Bonferroni's F, there were significant differences between the type I and type III computers; and between the type II and type III computers. Within the group III, when the type I computer was used, the mean reaction time was 619.2 ± 96.2 msec; when the type II computer was used, 587.1 ± 74.5 msec; and when the type III computer was used, 510.8 ± 58.5 msec. The difference among the three types of computers was significant (*p* < 0.001), and in the post hoc test by the Bonferroni's F, there were significant differences between the type I and type III computers; and between the type II and type III computers.

But in the group I, there was no significant difference in the mean reaction time among the three types of computers.

In addition, symbol digit, and finger tapping speed, there were no significant differences in the mean reaction time, standard deviation of the mean reaction times, the numbers of errors, and the numbers of tapping among the three types of computers (Tables 4 and 5).

## Discussion

The neurobehavioral test can be used in the occupational and environmental medicine for diagnosis and treatment of workers' neurological disorders including a follow-up of recovery patterns of the nervous system or disabilities after treatment, and for interruption of exposure to the suspected neurotoxic materials. Additionally, it has been used in neurotoxicological evaluation of hazardous substances or epidemiological studies for workers to set a limit value, etc. In particular, since 2009, as a secondary optional test in the special periodic medical examination for Korean workers, the KCNT has been used [17]. The advantages of the KCNT are as follows: the test method is standardized and simple; results are produced rapidly; the results are less biased by an examiner; storing the data is easy; and it has a higher compatibility.

However, the stimulus-reaction method is limited to the monitor and keyboard, thus low familiarity with the computer might affect the test performance [12, 18–20]. In other words, when the KCNT is performed, it is considered that the factors most difficult to adjust include subject's repulsion to the computer and differences in computer skills and capabilities. The factors may vary depending on the parameters of the KCNT and subject's characteristics. Therefore, they can be error factors reducing the validity of the KCNT [21, 22], where Jeon et al. [10] emphasized the importance of reducing the effect of the keyboard used as a tool for input in order to minimize the repulsion to the computer, lack of skills, etc. In the KCNT, the speed to find and press a specific key of using keyboard may be affected by computer familiarity. And if a simplified keyboard is used, which is made by removing the keys affecting the test from the typical keyboard, the influence of visual scanning on the keyboard can be reduced, and more valid results can be obtained by decreasing the influence of familiarity with the keyboard.

Moreover, another disadvantage of the KCNT is that computers need to be moved and installed for the test [10, 15]. Therefore, for the KCNT, a portable laptop computer can be utilized instead of a practically immobile desktop computer. Kim et al. [15] investigated whether there was a difference depending on the three types of computers (laptop computer alone, laptop computer equipped with a simplified keyboard, and desktop computer equipped with a simplified keyboard) and reported the decrease of validity due to the use of a laptop computer in the neurobehavioral test results of each parameter. They reported that for the parameters such as simple reaction time or finger tapping speed, a test could be conducted using a laptop computer only.

Meanwhile, the household use of a laptop computer in Korea increased remarkably as well as in workplaces compared to that in 2004 when the previous study on the validity of the KCNT was conducted. And people have become more familiar with the use of the computer over the last decade [16]. Such changes may affect the performance of KCNT. Therefore, it is important to re-evaluate the performance of KCNT depending on the familiarity and type of computer to suggest a methodologically up-to-date guideline.

Although there is no report on how to measure computer familiarity directly and objectively, Jeon et al. [10] used the Korean typing speed as an indirect parameter to classify subjects by computer familiarity. With this method, it can be assumed that computer familiarity has substantially increased since 2004 because the group I, defined as subjects with near-zero Korean typing speed, accounted for only 9.4 % of the subjects in this study, whereas as many as 34.3 % of the subjects were classified as group I in 2004. In addition, although the mean age of the group III composed of subjects who are highly familiar with the computer in this study is similar to that in 2004, the mean Korean typing speed increased from 224.2 per minute in 2004 to 325.4 per minute in this study. In the group II, although the mean age increased in this study compared to that in 2004, the mean Korean typing speed increased from 79.2 per minute in 2004 to 119.7 per minute in this study. A selection bias may have played a role in this change, but it is more plausible to assume that the proportion of persons who know how to use a computer have increased in general. Based on these facts, it can be assumed that examinees have been more familiar with the computer.

Jeon et al. [10] reported that the outcome of symbol digit was affected by computer familiarity, where the higher computer familiar group showed significantly shorter reaction time compared to the unfamiliar group. And in terms of simple reaction time, addition, and finger tapping speed, they were reported as parameters not affected by computer familiarity. Unlike the results of Jeon et al., the mean reaction time of symbol digit was not affected by computer familiarity; and with increasing computer familiarity, the standard deviation of mean reaction times of choice reaction time and symbol digit decreased, and the tapping number of finger tapping speed of the dominant hand increased. Therefore, the choice reaction time, the symbol digit, the number of errors in addition, and the finger tapping speed of the dominant hand were affected by computer familiarity, whereas the simple reaction time and the finger tapping speed of the non-dominant hand were not.

Jeon et al. [10] also reported that the reaction time of symbol digit decreased significantly and recommended the use of a 17-key simplified keyboard to exclude the effect of keyboard related familiarity [23]. However, in this study, there was no significant difference in symbol digit between the test results produced by the typical keyboard and by the simplified keyboard. Namely, in case of symbol digit for which examinees have to find a specific key and react, its mean reaction time was expected to be significantly shorten if the type of keyboard was simpler, but actually there was no difference between the mean reaction time of symbol digit by both types of keyboard in this study. And in this study, the type of keyboard did not affect the results of addition and finger tapping speed, which was similar to that of Jeon et al. [10]. Whereas there was significant difference in simple reaction time between the test results produced by both types of keyboard. The KCNT performance was affected by the keyboard even in case of the parameters of KCNT for which a specific key on the keyboard does not need to be scanned, which was not consistent with those of previous studies [10]. This finding is important that this means the simple reaction time, the most frequently used test, may also be influenced by the type of keyboard.

Meanwhile, the mean reaction time of choice reaction time was also significantly reduced when a simplified keyboard was used, particularly in the group II and the group III. Choice reaction time was not a parameter evaluated in Jeon et al. [10], and it is considered as a more sophisticated test than the simple reaction time since four arrow keys are used in the choice reaction time in comparison to the simple reaction time where only one key is used. In such cases, there is a higher chance of false positive results with a full-key keyboard and, thus, the results should be carefully interpreted.

Therefore, three parameters such as addition, symbol digit and finger tapping speed are not affected by the type of keyboard, whereas it is recommended to use simplified keyboard when performing the simple reaction time and the choice reaction time.

Types of computer may also affect the performance of KCNT as shown in the study of Kim et al. [15]. They reported that it requires extra caution when interpreting the result of symbol digit tested using a laptop computer because its reaction time was significantly shorter with a desktop computer equipped with a simplified keyboard compared to that with a laptop computer alone. However, the results of symbol digit did not show a significant difference between different types of computers in this study. Also, according to the study of Kim et al. [15], the simple reaction time and the choice reaction time were classified as tests that could be performed regardless of the type of computers. But in this study, because the performance of these parameters were better with a laptop computer equipped with a simplified keyboard and their reaction times were significantly shorter with a desktop computer with simplified keyboard, careful interpretation of tests performed with a laptop computer alone is suggestive.

In the remainder parameters such as addition, symbol digit, finger tapping speed, the performance of KCNT by different types of computer showed no significant difference. Therefore, laptop computer is not recommended for the simple reaction time, as well as for the choice reaction time in groups II and III. Such conclusion is not consistent with Kim et al. [15] who reported that laptop computer may be used for the mean reaction time of simple reaction time. Because workers today have an easier access to laptop computers than those 10 years ago, a laptop computer alone seems to be sufficient to perform the KCNT instead of a desktop computer equipped with a simplified keyboard in some parameters of KCNT. The outcome of the simple reaction time and choice reaction time, however, were not valid with a laptop computer alone, which means it is most appropriate to use a desktop computer equipped with a simplified keyboard for these tests.

The limitations of this study include small sample size and the composition of study subjects. The sample size was calculated based on the simple reaction time, which is the most frequently used parameter of the KCNT, and was sufficient in general, but the number of subjects in each group was relatively small. If a study is performed with a larger number of subjects, the statistical power will be increased. And the age and level of education were not controlled in the subject selection process but in a statistical way, the differences in age and the level of education among groups could not be removed.

## Conclusions

Among the parameters of the KCNT, the parameter most affected by the computer familiarity and the type of keyboard was symbol digit in the study of Jeon et al. [10], but now 10 year later the simple reaction time, choice reaction time, the number of errors in addition, and finger tapping speed (dominant hand) were affected. Therefore it is recommended to select the parameter such as addition, symbol digit, and finger tapping speed (non-dominant hand) to assess the neurobehavioral performance of the workers with low computer familiarity. Moreover, among the parameters of KCNT, the parameters that could be tested using a laptop computer included simple reaction time and finger tapping speed in the study of Kim et al. [15], but now 10 years later, addition, symbol digit, and finger tapping speed could be performed only using a laptop computer. Additionally, in order to obtain the valid results, before the KCNT, it was recommended that the appropriate parameters should be selected after computer familiarity is checked based on the Korean typing speed, etc. Moreover, if a laptop computer is used inevitably, a laptop computer equipped with simplified 17-key keyboard should be used.
